# Shattered Dreams: Dysfunctional Families and Suicidal Behaviour among Armenian Adolescents

**DOI:** 10.1192/j.eurpsy.2025.721

**Published:** 2025-08-26

**Authors:** M. Arsenyan

**Affiliations:** Department of Child Psychiatry, ‘Surb Grigor Lusavorich’ Medical Center, Yerevan, Armenia

## Abstract

**Introduction:**

The family is the cornerstone of a child’s physical, emotional, and psychological development (Masteller & Stoop. Servant Pub. 1996; Ubaidi. J Fam Med Dis 2017; 3:059). While a stable family fosters healthy growth and positive interpersonal relationships, a dysfunctional family can lead to severe consequences, especially in adolescence (Kurtz & Derevensky. Can. J. Sch. Psychol 1994; 9(2) 204-216; Holland *et al*. J. Youth Adolesc. 2017; 46(7) 1598–1610). In Armenia, where domestic violence is widespread and underreported due to societal norms prioritising family unity and reputation preservation (Kharatyan & Tovmasyan. Eur. J. Soc. Sci. Educ. Res. 2021; 8(1) 56-65), research on the effects of dysfunctional family dynamics on adolescent suicidality remains notably scarce.

**Objectives:**

This study explores the role of dysfunctional family structures in suicidal behaviour among adolescents in Armenia.

**Methods:**

A qualitative analysis employing the documentary method was conducted on the patient histories of 67 adolescents hospitalised at ‘Muratsan’ University Hospital Complex following suicide attempts.

**Results:**

Of the 67 adolescents, 30 had divorced parents, and 37 had intact families. Five key themes—pathological, authoritarian, conflict-driven, chaotic, and neglectful family environments—emerged, highlighting the development of adolescent suicidal behaviour. Adolescents from pathological families, where one or both parents struggled with substance abuse or mental health conditions, frequently witnessed and experienced ongoing domestic violence. In authoritarian families, adolescents were subjected to coercive and controlling behaviour, while those from conflict-driven families were often caught in intense interpersonal strife or between feuding, divorced parents. In chaotic families, parents were unable to meet the adolescents’ emotional needs due to their overwhelming focus on financial responsibilities and coping with the fallout of divorce. In neglectful families, adolescents experienced a profound lack of parental involvement and support, often feeling abandoned. These dysfunctional family environments fostered suicidal behaviour, with adolescents attempting suicide as they ‘couldn’t bear their situation any longer’, wanted to ‘escape reality’, ‘couldn’t see a way out’, ‘didn’t feel needed by their parents’, ‘couldn’t find a reason to live’ and ‘wished to die’.

**Conclusions:**

The study revealed that adolescent suicidality arose within dysfunctional family environments characterised by ongoing exposure to domestic violence, coercive and controlling behaviour, conflicts, parental neglect, and a lack of emotional support. These findings highlight the critical need for targeted interventions to support at-risk youth, emphasising the urgency of addressing domestic violence against women and children in Armenia and assisting parents in fostering a caring and safe family environment.

**Disclosure of Interest:**

None Declared

